# Can brain impermeable BACE1 inhibitors serve as anti-CAA medicine?

**DOI:** 10.1186/s12883-017-0942-y

**Published:** 2017-08-25

**Authors:** Jian-Ming Li, Li-Ling Huang, Fei Liu, Bei-Sha Tang, Xiao-Xin Yan

**Affiliations:** 1 Department of Neurology & Key Laboratory of Hunan Province in Neurodegenerative Disorders, Xiangya Hospital, Central South University, Changsha, Hunan 410008 China; 2 Neuroscience Research Center, Changsha Medical University, Changsha, Hunan 410219 China; 3 Department of Anatomy and Neurobiology, Central South University School of Basic Medical Science, Changsha, Hunan 410013 China; 4 Department of Neurosurgery, The Third Xiangya Hospital, Central South University, Changsha, Hunan 410013 China

**Keywords:** Alzheimer’s disease, ß –Amyloid, Cerebral amyloidosis, Neurodegeneration, Vascular dementia

## Abstract

**Background:**

Cerebral amyloid angiopathy (CAA) is characterized by the deposition of ß-amyloid peptides (Aß) in and surrounding the wall of microvasculature in the central nervous system, together with parenchymal amyloid plaques collectively referred to as cerebral amyloidosis, which occurs in the brain commonly among the elderly and more frequently in patients with Alzheimer’s disease (AD). CAA is associated with vascular injury and may cause devastating neurological outcomes. No therapeutic approach is available for this lesion to date.

**Main body:**

ß-Secretase 1 (BACE1) is the enzyme initiating Aß production. Brain permeable BACE1 inhibitors targeting primarily at the parenchymal plaque pathology are currently evaluated in clinical trials. This article presents findings in support of a role of BACE1 elevation in the development of CAA, in addition to plaque pathogenesis. The rationale, feasibility, benefit and strategic issues for developing BACE1 inhibitors against CAA are discussed. Brain impermeable compounds are considered preferable as they might exhibit sufficient anti-CAA efficacy without causing significant neuronal/synaptic side effects.

**Conclusion:**

Early pharmacological intervention to the pathogenesis of CAA is expected to provide significant protection for cerebral vascular health and hence brain health. Brain impermeable BACE1 inhibitors should be optimized and tested as potential anti-CAA therapeutics.

## Background

Cerebral amyloid angiopathy (CAA) refers to ß-amyloid (Aß) deposition in and surrounding the wall of cerebral vasculature, often involving small to mid-sized arteries, and less commonly capillaries and veins. Aß deposition along the leptomeninge is also considered a part of CAA [1–5]. Aging and Alzheimer Disease (AD) appear to be the major risk factors for CAA. Epidemiological studies suggest that 10% to 40% of the elderly have CAA, with the frequency raised up to 80% among patients with AD [6]. The incidence of moderate to severe CAA ranks approximately 2.3%, 8% and 12.1% among individuals at 65–74, 75–84 and over 85 years of age, respectively [1, 7]. Compared to non-demented individuals, the morbidity and severity of CAA both appear to be increased in demented or AD subjects. Thus, although CAA may be considered as a sign of brain aging, it could be related to the development and progression of dementia of the AD and vascular types [8–14].

While CAA is considered as a pathological change than disease entity, its clinical implication has gained growing attention in the medical field. CAA appears to be one of the most common reasons for primary, non-traumatic and non-hypertensive cerebral haemorrhage [4, 5, 10, 15]. Elderly with mild CAA in their brains might exhibit no neurological symptoms. With the progress of CAA, more damage and breakdown of the blood-brain barrier (BBB) and vascular wall can occur, raising the risk of suffering from overt clinical symptoms possibly as a result of silent but substantial intracranial haemorrhage and ischemic neuronal stress and injury [10, 13, 16–18]. Unfortunately, there are no preventive or therapeutical approaches available for CAA to date [19].

Brain imaging technologies are advancing quickly and can nowadays detect signs of CAA at preclinical stages [20–22], providing potential screening guide for early pharmacological intervention to the lesion among at-risk individuals. Progress in basic and pathological research has been also made in understanding of the pathogenesis of CAA. Specifically, recent studies have extended evidence in support of an involvement of BACE1 elevation in CAA pathogenesis [23–25], in addition to amyloid plaque formation. This raises an opportunity of using BACE1 inhibition as a therapeutic, perhaps even preventive, option to delay or slow-down the development of CAA and thus mitigate its destructive neurological consequences. While BACE1 inhibition is being vigorously explored in clinical trials as an anti-Aß therapy primarily targeting at the parenchymal plaque lesions, there is less discussion about its potential for the treatment of CAA. In this review, we first briefly introduce the biochemical aspects of Aß genesis and clearance, and the cellular expression of Aß-producing proteins in the brain including vasculature, with a preference given to update BACE1-related data. We then address the pathological and pathogenic aspects of CAA, focusing on recent findings about the role of BACE1-mediated Aß overproduction. Finally we discuss the benefit, feasibility and some strategic issues for developing BACE1 inhibitors primarily targeting at CAA, in addition the compounds designated to reduce amyloid plaque lesions explored currently in clinical trials. Given the interconnecting nature of CAA with parenchymal amyloidosis, issues related to the amyloid plaque pathology and its intervention are also covered briefly while addressing the above topics.

## Main text

### Biochemical perspectives of Aß production and clearance

ß-Amyloid peptides are derived from the ß**-**amyloid precursor protein (APP), which is an integral membrane protein ubiquitously expressed in cells of the body including neurons [26–28]. APP can interact with many adaptor proteins and bind to some extracellular matrix components including heparin and collagen, as such serving a crucial role in cell-cell communication and intracellular signalling. APP may be involved in broad biological functions in the body, including hormonal regulation [29] and iron export [30], and in the nervous system, participates in neuronal development, signal transduction, axonal transport, synaptic formation and repair [31–37].

Biochemically, APP is cleaved by the so-called secretases, and by some other proteolytic enzymes as well, yielding many forms of fragment products [38–42]. The secretase-mediated cleavages include the non-amyloidogenic and amyloidogenic pathways. The former is executed by α-secretase and γ-secretase complex, likely as the predominant form of APP processing under physiological condition. The amyloidogenic pathway is initiated with ß-site cleavage of APP by BACE1, releasing ß-site cleaved C-terminal fragments (β-CTFs), which are further cut by γ-secretase to produce Aß [43–47]. Depending on the sites of γ-cutting, Aß may contain amino acid (a.a.) residues of varying lengths [48]. The Aß mono-peptides could bind together to form soluble oligomers, and insoluble aggregates that deposit as microscopically evident extracellular lesions in the brain. A new η-secretase APP processing pathway initiated by some membrane-bound matrix metalloproteinases has been identified lately, which may lead to the formation of long and short Aη peptides as end products following α- and ß-secretase proteolyses [49].

Besides regulation at the level of production, several clearance mechanisms help maintain the concentration of Aß in the brain homeostatically. Thus, it is suggested that Aß products are removed from the brain through (1) being endocytosed by astrocytes and microglia; (2) being degraded by some enzymes including neprilysin and the insulin-degrading enzyme; (3) being cleared from brain parenchyma into blood after passing through BBB; (4) being drained out of the brain through the periarterial spaces via some specific routes, for instance, along intracortical microvascular walls to leptomeningeal arterial walls, and finally to cranial and cervical lymph nodes [50–53].

### Cellular localization of Aß-producing proteins in the brain including vasculature

Many original studies and reviews have described the cellular expression pattern of the Aß-producing machinery, i.e., APP, BACE1 and subunit proteins of the γ-secretase complex, in mammalian brains e.g., [54, 55], therefore we only shortly note this issue here. Overall, neurons appear to be the major cell type in the brain with enriched expression of APP and BACE1, as well as presenilins (PS1 and PS2) that serve as the catalytic core of γ-secretase complex. Previous studies have shown distinct immunolabeling of APP and PS1/PS2 in the somata and dendrites of neurons in mammalian brains [56–58]. Immunolabeling of BACE1 has been detected in neurons and glial cells [59], while some antibodies label a pattern with predominant reactivity in the neuropil, particularly distinct at some brain areas rich of synaptic terminals, i.e., the olfactory bulb glomeruli and hippocampal mossy fibres [60–64]. The notion that presynaptic terminals as an important site of Aß production is supported by increasing cell biology, biochemical and anatomical data [65–71]. Specifically regarding synaptic function and plasticity, BACE1 is shown to be dynamically transported in axons via antegrade and retrograde trafficking [65, 66]. Moreover, APP cleavages can occur directly in synaptic vesicles, with Aß produced at and released by presynaptic axon terminals [67–71].

Apart from neurons, many studies have demonstrated that the cellular components of blood vessels possess the biochemical machinery for Aß production. At the messenger level, vascular endothelial cells express three alternatively spiced APP mRNA isoforms, APP695, APP751 and APP770, in comparison with neurons that appear to only or predominantly express APP695 [72]. In cell culture studies, brain microvascular endothelial cells and human umbilical vein endothelial cells are found to express APP, BACE1 and PS1 by immunoblot and immunocytochemical analyses, and can secret Aß into culture medium detectable by ELISA [73–77]. Similar results are obtained from experiments using primary cell cultures prepared from surgically removed human blood vessels [25]. Some studies have also shown that cultured vascular smooth muscle cells express APP, BACE1 and PS1 [23, 78, 79]. Notably, in histological preparations of animal or human brains without amyloid pathology, the cerebral blood vessels or their cellular components (i.e., endothelial and smooth muscle cells) generally do not exhibit impressive immunolabeling of APP, BACE1 or PS1, relative to the immunoreactivity seen in neuronal elements [56, 57, 60–62, 80]. Thus, the overall levels of expression of the Aß producing proteins in vascular cells are apparently lower relative to neurons in normal animal or human brain.

### Pathological characteristics of CAA

As with the amyloid plaque lesions, CAA can occur broadly in the central nervous system including the spinal cord [1–7, 81]. In comparison, little information indicates the existence of parenchymal and angiopathic types of Aß deposition in the periphery [82]. Such an organ/tissue preference of ß-amyloidosis appears to suggest some root link of the lesions to specific cellular components of the nervous system. Overall, cortical and leptomeningeal arteries appear to be mostly susceptible to CAA, while Aß deposition could occur at veins and venules. The neocortical vessels appear to be firstly and most frequently affected, followed by that in the paleocortex and midbrain. Less involved areas include the basal nuclei, dorsal thalamus and brainstem [1–8, 83–85].

**Fig. 1:**
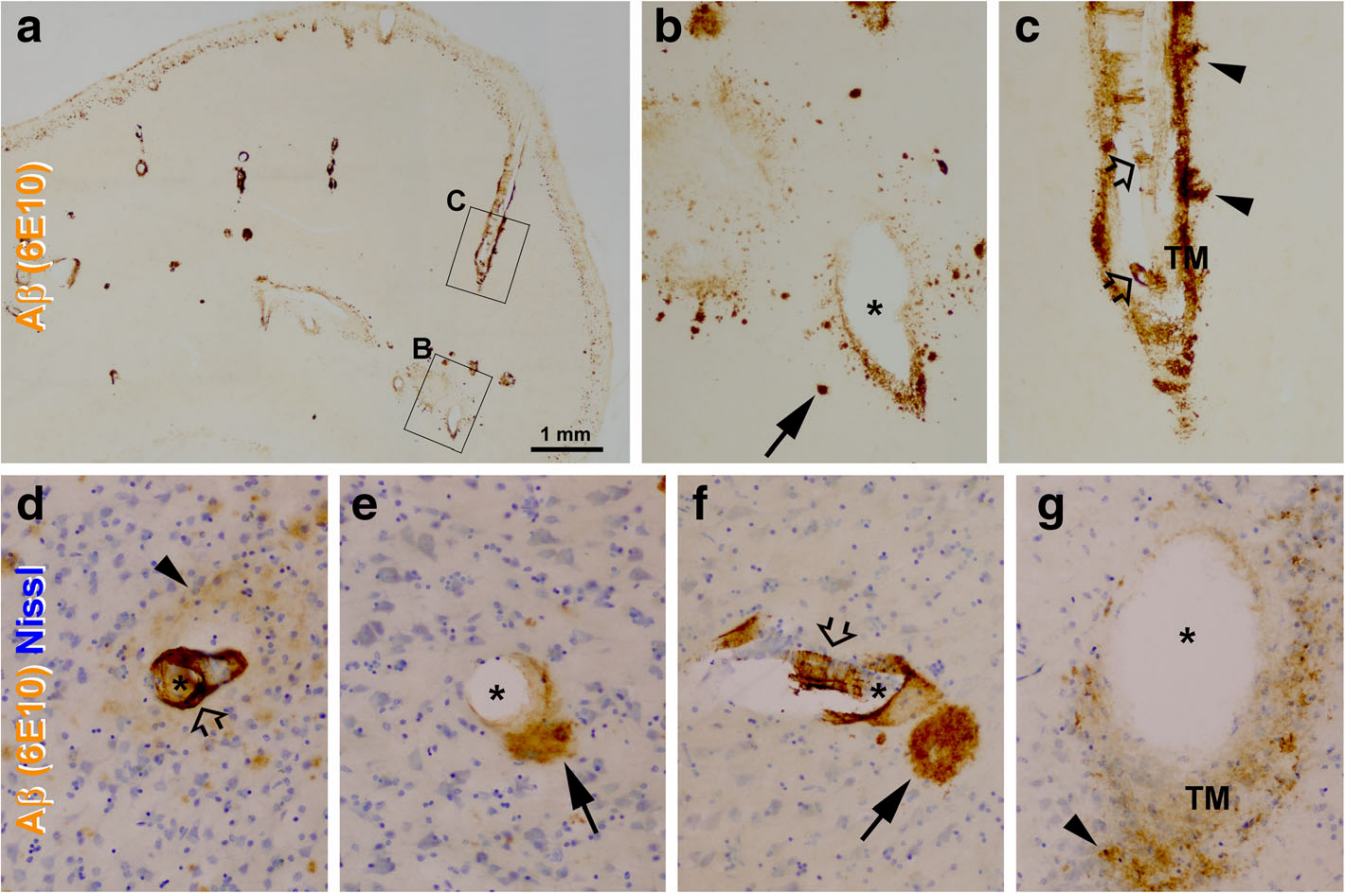
Microscopic images showing examples of cerebral amyloid angiopathy (CAA) from aged human brain. Panel (**a**) is a low magnification view of β-amyloid (Aβ) immunolabeling with the monoclonal antibody 6E10 obtained from the temporal neocortex. Meningeal amyloid labeling is present along the cortical surface, with labelled individual arterioles of varying sizes invading and inside the cortex. Punctuate immunoreactive profiles occur along the low portion of layer I. Panels (**b**) and (**c**) are enlarged views of the framed areas in (**a**). Panels (**d**-**g**) are high power views of 6E10 immunolabeling with Nissl counterstain. Note the presence of perivascular amyloid deposition (pointed by arrows and arrowheads) in addition to the labeling in the wall of affected vessels, which appear diffuse (arrowheads) or compact (arrows) like in morphological pattern. Both large (**c**) and small (**d**, **f**) vessels can exhibit heavy immunolabeling at the inner layer of the vascular wall (open arrows). *: vascular cavity. Scale bar = 1 mm in (**a**), equal to 200 μm for other panels. Images are adapted from original data of recent studies [25, 162]

**Fig. 2:**
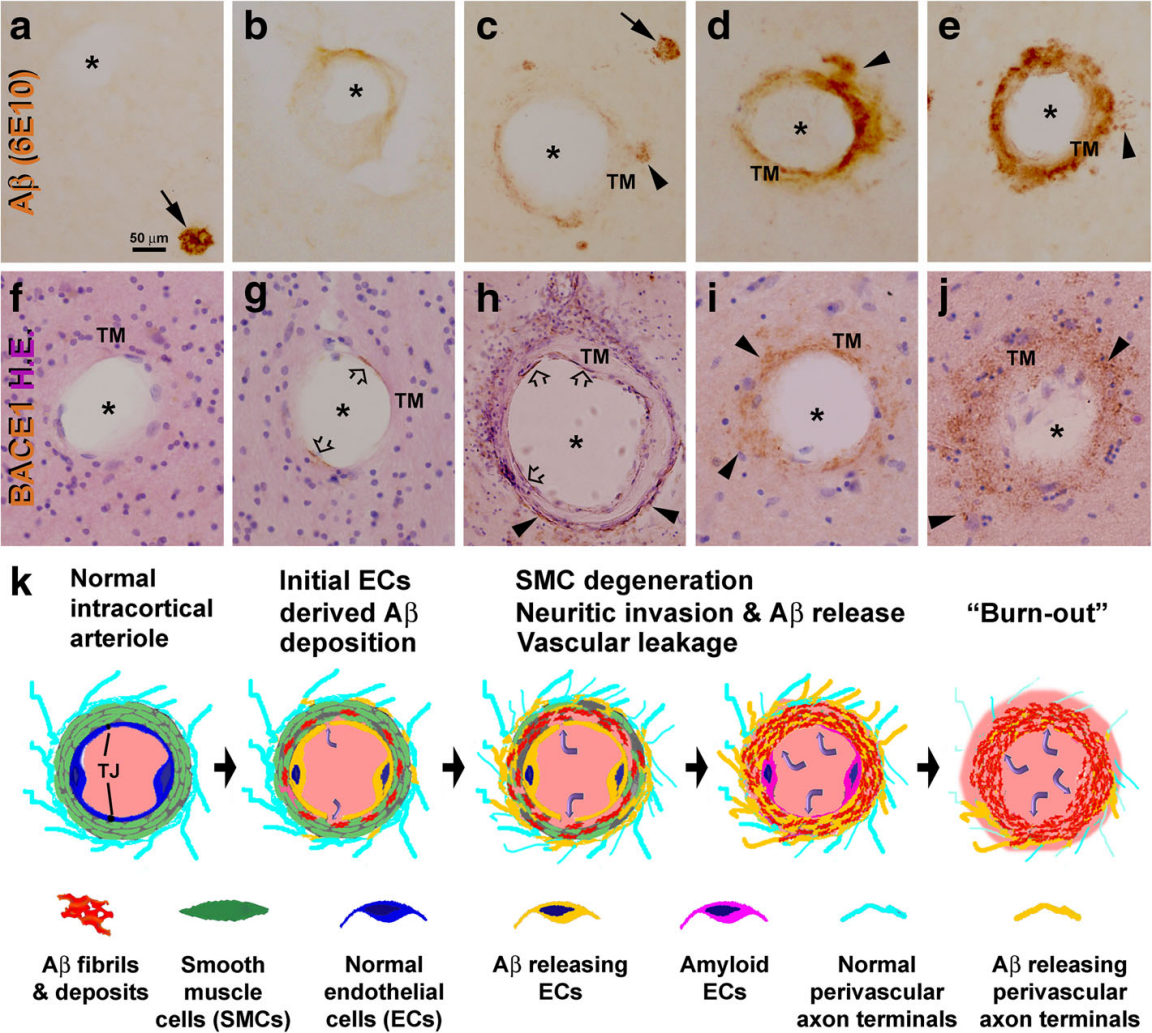
Microscopic images and schematic drawings illustrating a hypothetic model of cerebral amyloid angiopathy. Panels (**a**-**f**) show arterioles without (**a**) and with β-amyloid labeling of increasing intensity. Aβ deposition appears to occur around the tunica intima or endothelial layer (**b**, **c**), then emerges and accumulates in the tunica media (TM) (**c**-**f**). Perivascular deposition (arrowheads) is seen around vessels with mild to severe Aβ labeling in the vascular wall (**c**-**f**). Panels (**f**-**j**) are images of β-secretase 1 (BACE1) immunolabeling with **h**.**e**. counterstain, showing arterioles without (**f**) and with immunolabeled elements exhibiting a progressive pattern (**g**-**j**). Thus, BACE1 labeling is identifiable at the endothelial layer in (**g**, **h**), occurs at the perivascular zone in (**h**) and presents across the vascular wall and perivascular zone in (**i**, **j**). Arrows point to compact plaques. Scale bar = 50 μm in (**a**) applying to (**b**-**g**, **i**, **j**), equal to 100 μm for (**h**). Panel (**k**) illustrates a hypothetic model for BACE1 elevation in vascular and brain-specific cellular elements in the development of CAA. BACE1 elevation first occurs in endothelial cells (ECs), resulting in Aβ accumulation in the smooth muscle cell (SMC) layer. This causes damage of tight junctions (TJ) and leakage of blood contents into the SMC layer (curved arrows). Aβ and blood infiltration then induce SMC degeneration, triggering aberrant sprouting of the perivascular axonal terminals inherent neuronal Aβ overproduction. This process continues progressively and may end up with a “burnout” stage whereby the ECs, SMCs and dystrophic neurites all degenerate. This figure is adapted with modification from Fig. 7 in [25]

The onset and progression of CAA at a given vascular site are still not well understood to date. Pathological observations suggest that at arterioles Aß products may first deposit in the basement membrane of the tunica intima, then accumulate in the smooth muscle layer or the tunica media. The smooth muscle cell layer may be eventually destroyed and replaced by Aß fibrils, which could mount progressively to become very heavy to a point until no cellular components in this layer left [86]. Notably, perivascular Aß deposition can occur along with the lesion inside the vascular wall. The profiles with concurrent vascular and perivascular Aß deposition have been defined as “dysphoric” or “dyshoric” CAA [86–89]. We observed fairly frequent perivascular Aß deposition at cerebral vasculature with either mild or severe amyloidosis inside the wall or the smooth muscle cell layer in postmortem human brains [25] (Fig. 1a-c). Some perivascular Aß deposition exhibited the morphological configuration of diffuse or compact plaques (Fig. 1d-g). When comparing cortical arterioles with varying extent of Aß deposition, a pattern of pathological evolution appeared to exist. Thus, Aß immunolabeling appeared to occur initially at the tunica intima, followed by the tunica media and perivascular zone. In some arterioles and capillaries, the tunica intima or endothelial cells exhibited extremely heavy Aß immunolabeling (Fig. 2a-f).

### Role of Aß overproduction in parenchymal plaque amyloidosis

The pathogenic mechanism underlying cerebral amyloidosis as either parenchymal plaques or CAA is still not consensually defined, with the lesions collectively regarded as resulted from an imbalance between the production and clearance of the Aß peptides. It is suggested that a global rise of Aß levels in the interstitial fluid of the brain triggers self-propelling aggregation of the peptides to form insoluble products. According to the original amyloid hypothesis [90], reduced Aß clearance from the brain is the principal or primary causal factor for cerebral amyloidosis in sporadic AD. Among the Aß clearance mechanisms as noted above, the blocking of perivascular drainage is considered a crucial one. It is also suggested that Aß42 tends to precipitate in brain interstitial space to form senile plaques, whereas Aß40 has a better solubility, therefore more likely to be drained away through the perivascular space and thus deposit preferentially at cerebral vessels as CAA [16]. Increased peripheral Aß transit across the blood brain barrier (BBB) and “back-flushing” into the brain is also hypothesized [91–97]. Of note, recent studies have shown that reducing periphery Aß does not apparently alter brain amyloidosis or Aß levels in the cerebrospinal fluid [98–100]. Increased Aß42/Aß40 ratio [16, 100–103] and prion-like propagation [104–107] are also suggested to promote cerebral amyloidosis. Overall, an unifying hypothesis remains to be established to coherently explain as to why Aß deposition manifests in different patterns, occurs at selected locations and develops site-specifically in the human brain.

Data are also collected pointing to a role of localized Aß overproduction in cerebral plaque formation. Increased BACE1 expression and activity are reported in the brains from individuals with sporadic AD [108–116]. To address the issue as to whether BACE1 elevation may occur in spatial relevance with parenchymal Aß deposition, several groups used well-characterized BACE1 antibodies for comparative analysis of BACE1 and Aß immunolabeling in the brains of transgenic AD mouse models, aged humans and clinically diagnosed AD subjects, and aged nonhuman primates [62, 80, 113–116]. These studies have consistently demonstrated increased BACE1 expression in swollen/sprouting axonal terminals, commonly referred to as the dystrophic neurites, in neuritic plaques.

Whether chronic/persistent BACE1 elevation in association with an ongoing neuritic pathogenesis plays a principal role in plaque formation in the human brain remains to be established. The amounts of BACE1 labelled dystrophic neurites do not always match to the extent of amyloidosis anatomically and densitometrically in postmortem human brains, with Aß deposition appeared much denser relative to neuritic profiles especially among the cases with advanced AD pathologies [80]. However, one can argue that the two measurements should not match to each other for several reasons. First, Aß immunolabeling seen in the sections would most likely represent the amount of insoluble deposits accumulated over time, while BACE1 immunolabeling would more likely reflect the amount of the enzyme present at the cross-sectional time point when the brain is processed. Second, insoluble Aß deposits are known to resistant to postmortem tissue/protein degradation. In fact, amyloid pathology can been detected in human brains collected months even years after death [117–119]. On the other hand, histological integrity and biological molecules including normal neural proteins are decomposed rapidly if brain samples are not preserved by tissue fixation on time [117]. Moreover, dystrophic neurites of the amyloid plaques show high structural plasticity over time [120], and Aß production as a biochemical process is depended on the viability of the dystrophic neurites [121]. With the build up of Aß deposits, viable cellular elements including dystrophic neurites may be dying out because of the so-called “burn-out” effect [122, 123].

For the other type of parenchymal plaques, the diffuse plaques, it has been difficult to determine if BACE1 elevation in local cellular elements is involved. When examining human brain samples, no distinct profiles with enhanced BACE1 immunolabeling are detectable over the areas with diffuse amyloid deposition (own experience). This is also the case for the fairly distinct Aß labeling along the low portion of cortical layer I as seen in some human brains (as shown in Fig. 1a for example). Layer I is innervated richly by axonal terminals from some subcortical structures, including the monoaminergic projections from the brainstem [124, 125]. These fine axonal terminals also distribute along the wall of pial arteries invading into the cortex, serving a key role in coupling neuronal activity and blood supply. Notably, in examination of perfusion-fixed brains from some transgenic mouse models of AD, e.g., the 3×Tg-AD mice, increased BACE1 immunolabeling at fine axonal profiles are clearly present over the areas with diffuse Aß deposition in the forebrain [126].

In the human or transgenic AD mouse brains wherein the neuropathology is well established, it is difficult to determine the causal relationship between axonal dystrophy and Aß deposition as one attempts to address the issue of onset and development of the neuritic plaques. A dynamic interplay between the two would likely exist, resulting in a feed-forward vicious cycle in the course of plaque pathogenesis [55, 66, 117]. Notably, in wild-type animals wherein no elevated Aß levels are pre-existed in the brain, many experimental insults, such as traumatic brain injury [127–130], microvascular embolism [131], pilocarpine-induced temporal lobe epilepsy [132] and endotoxin-induced neuroinflammation [133], can clearly induce a dystrophic axonal pathology inherent with APP/BACE1 upregulation in the affected brain regions, although extracellular Aß deposition may not be detectable. When such experimental insults are applied to transgenic AD models, they could accelerate the age-related development of neuritic plaque pathogenesis in the brain [134–136].

### Role of Aß overproduction in cerebral angiopathic amyloidosis

Many groups of investigators have carried out in vitro and in vivo experiments to explore a potential involvement of Aß overproduction in the development of CAA [23–25, 73–78]. Specifically, an active role for BACE1-mediated Aß overproduction in vascular endothelial cells has been proposed for angiopathic amyloidosis [23–25]. We examined the spatial relationship between BACE1 expression and CAA in postmortem human brains, with particular attention paid to understand the cellular components potentially exhibiting increased BACE1 immunolabeling around amyloid vasculature [25]. There appeared to exist a comparable pattern between Aß and BACE1 immunolabeling in and surrounding the wall of cortical arterioles (Fig. 2a-j). Enhanced BACE1 immunolabeling around the tunica intima appeared to occur in the endothelial cells (Fig. 2g, h). BACE1 immunoreactive profiles were also found in the tunica media and the perivascular regions, identified as neuronal processes rather than smooth muscle cells, neurons or glial cells (Fig. 2h-j). These BACE1 labelled profiles represented sprouting axonal profiles given their colocalization with synaptophysin, and nicotinamide adenine dinucleotide phosphate-diaphorase (NADPH-d) that is expressed in the processes of nitric oxide (NO) producing interneurons [137]. Based on these observations, we proposed a dual-origin model for angiopathic Aß deposition in the human brain [25] (Fig. 2k). Thus, vascular endothelial cells begin to over-express BACE1 (likely APP as well) and increase Aß production in response to certain circulatory stress factors. The Aß products are released into blood (hence cleared away) as well as inside the vascular wall, with the latter retained and deposited locally causing some initial damage of the tight junction between endothelial cells. The accumulation of Aß together with infiltration of blood (potentially containing inflammatory factors) resulted from BBB leakage into the vascular wall causes stress/damage and degeneration of the smooth muscle cells. Cellular toxicities from Aß and other inflammatory molecules as well as a space-emptying effect in the smooth muscle cell layer may trigger a regenerative response of the perivascular axonal terminals, manifested by aberrant sprouting into and surrounding the vascular wall. Because the axonal pathology is inherent with BACE1/APP overexpression, a second wave of increased local Aß production occurs and mounts Aß products on-site. This neuronal contribution of Aß could continue until some point whereby all viable cellular components (vascular cells and dystrophic neurites) become degenerated (“burn-out”), leaving the original vascular site as an end-pathology locus heavily filled with Aß fibrils. This dual-origin hypothesis could explain as to why CAA can occur in and surrounding the wall of cerebral arterioles, capillaries and venules, although the latter two have no or only a thin small muscle cell layer. It can also help understand as to why amyloid angiopathy is observed in the central nervous system yet rarely (if any) in the peripheral organs. It should be noted that the leptomeningeal amyloidosis might also develop via a dual or triple origin mechanism, such that vascular endothelia, perivascular axonal processes and the meningeal cells participate in Aß overproduction resulting in spreading amyloidosis along the cortical surface [25] (Fig. 1a).

Considering endothelia as an early CAA contributors, it would be important to explore if some circulatory factors may promote the amyloidogenic response of these cells. Epidemiological studies suggest that many cardiovascular and metabolic conditions are risk factors for dementia of the vascular and AD types, including hypertension, hypotension, hypercholesterolemia, atherosclerosis and diabetes [138–143]. In vitro studies have demonstrated that oxygen and glucose deprivation [75], proinflammatory cytokines [77] and high cholesterol [144] may serve as stress factors to stimulate endothelial Aß production possibly via BACE1 upregulation. The endothelial nitric oxide synthase (eNOS) could participate in modulating the amyloidogenic processing pathway in vascular endothelia [74].

### Brief update on BACE1 inhibitors targeting against the amyloid plaque pathology

The anti-Aß approach for the treatment of AD has been vigorously pursued in the past two decades. Overall, most drug candidates based on active and passive Aβ immunization, anti- Aß aggregation and γ-secretase inhibition have failed in clinical trials. BACE1 inhibition has been left as a highly expected anti-Aß option, although the final outcome of ongoing drug trials remains uncertain. In-depth reviews on the anti-Aß therapies for AD treatment/prevention are available, with some works focused on the BACE1 inhibition strategy specifically [54, 55, 145–152]. To avoid redundancy, we will only denote on a few issues here as the following.

Sufficient target engagement to neuronal BACE1 has been much concerned in the development of BACE1 inhibitors to minimize plaque pathology. As the catalytic core of the enzyme is relatively wide, early generations of BACE1 inhibitory compounds are too large to have sufficient brain penetration [149, 151]. With chemical modification of lead compounds, brain permeable BACE1 inhibitors have been successfully developed, with several front-running candidates in the pipeline entered clinical trials. LY2886721 is the first reached Phase II trail, which was discontinued due to potential liver toxicity (https://investor.lilly.com/releaseDetail.cfm?ReleaseID=771353). Verubecestat represents the latest BACE1 inhibitor with initial clinical trial data reported. This compound has excellent safety and target engagement profiles according to phases I and II trials, but has been also terminated in phase III trials among patients with mild to moderate AD at the time of subject registration, due to a lack of efficacy in slowing down cognitive decline (http://www.businessinsider.com/r-acceras-alzheimers-trial-fails-in-yet-another-setback-for-disease-2017-2). Currently, stage III trials on Verubecestat are continued in patients with early or prodromal stage AD, which might soon provide crucial assessment for the therapeutic efficacy of BACE1 inhibition (or anti-Aβ therapy in general) for AD.

However, potential side-effects of long-term BACE1 inhibition are a concern for the currently explored brain penetrant compounds. While initial data from BACE1 knockout mice suggest that BACE inhibition may not cause serious neurological side effects [54], it is now known that BACE1 plays critical biological roles in the central and peripheral nervous systems, including for axon growth and myelination, neuronal and glial genesis, ion channel activity and neuronal excitability [146, 148, 150–154]. Thus, BACE1 deficiency might cause impairment of physiological neuronal and synaptic functions. In fact, BACE1 null mice exhibit some neurological and cognitive deficits [60]. They also show malformation of the olfactory bulb glomeruli and axonal mis-targeting in the hippocampal mossy fiber pathway [60, 61, 63, 64]. More recent studies indicate that pharmacological inhibition of BACE1 with brain penetrant compounds can cause neuronal and synaptic deficits. For instance, prolonged treatment with the inhibitor SCH1682496 or LY2811376 can lead to reduced spine formation of layer V pyramidal neurons in adult mice. The rate of spontaneous and miniature excitatory postsynaptic currents in pyramidal neurons and hippocampal long-term potentiation are also reduced in the drug-treated animals. Moreover, cognitive deficits are observed in behavioural tests in these animals [155]. Overall, a relatively long period of evaluation would be needed to assure the safety of the brain penetrating BACE1 inhibitors.

### Benefit, feasibility and strategy for developing anti-CAA BACE1 inhibitors

As denoted in the preceding sections, CAA is associated with structural damage of the BBB and the entire vascular wall that can impair the regulated blood supply to brain parenchyma. Vascular leakage and micro-bleeding may also directly cause stress and damage to neuronal structures around the lesion [1–15]. Importantly, microvascular damage has been suggested to potentiate some neurodegenerative changes in AD, including the development of neuritic plaques because they are distributed in close proximity to capillaries [80, 88, 89, 131, 156–160]. Given the direct and indirect contribution of CAA to neuronal dysfunction and cognitive impairments during aging and in dementia of the vascular or AD type, therapeutic approaches to prevent or halt this pathology should be considered and pursued. Because CAA involves essentially the microvasculature, pharmacological intervention is likely the only solution. Although the development of CAA may have many upstream etiological factors, Aß accumulation and deposition are the core event or a final crossroad of the condition. Interrupting at this crucial step of CAA development appears to be well justified theoretically.

The experimental data suggesting a key role of BACE1 upregulation in the pathogenesis of CAA by mediating Aß overproduction in local cellular components, as assembled in our dual-origin hypothesis (Fig. 2k), highlight the feasibility for using BACE1 inhibitors as putative anti-CAA therapeutics. Vascular endothelia participate in the initial step of Aß overproduction, therefore confronting this alteration by BACE1 inhibition could provide early protection. Inhibiting endothelial BACE1 activity can be expected to reduce the release of Aß into the smooth muscle layer of arterioles and venules, therefore protect the integrity of vascular wall, and stop/delay vascular leakage and microhemorrhages. This would minimize the stress to perivascular neuronal processes and their reactive sprouting into and surrounding the vascular wall in association with neuronal Aß overproduction. In capillaries, BACE1 inhibition in endothelial cells may prevent these cells from becoming amyloid, and also prevent the pericapillary neuronal processes from undergoing amyloidogenic axonal pathology. In the case that a certain degree of CAA and vascular damage have already taken place, BACE1 inhibitors might get access into the smooth muscle cell layer and the perivascular zone, and target on neuronal BACE1 overexpression in the sprouting axonal processes. Thus, BACE1 inhibitors may be expected to target-engage with the cellular elements involved in the early as well as ongoing phases of Aß overproduction during the development of CAA.

In the pharmaceutical industry, there are examples that opportunity and success of drug discovery arise from initially unspeculated therapeutic target or effect of the candidate drugs (as was the case of Viagra). As mentioned earlier, during the development of BACE1 inhibitors many lead compounds are discarded from further translational evaluation because they are not brain penetrant to target neuronal BACE1 [149]. The discussions elaborated above would imply that brain impermeable inhibitors could otherwise serve as desired anti-CAA drug candidates. Without passing through the BBB, they may inhibit BACE1 activity in vascular endothelia therefore provide the first line protection against amyloid damage to blood vessels [24]. They may also diffuse into the vascular wall or to some extent the perivascular area in the case that some BBB breakdown has already occurred, which could still reduce the Aß genesis in the smooth muscle cell layer, brought in by the invading perivascular neuronal processes. In this regard, it is of interest to note that typical neuritic plaques are anatomically vasocentric since they occurs preferentially next to capillaries [80, 130, 156–158]. In addition, cellular and molecular components of blood are detected in neuritic plaques, suggestive of the existence of vascular leakage [159–165]. Thus, while brain impermeable BACE1 inhibitors are expected to primarily act against CAA, theoretically they can also diffuse across the damaged capillary wall and inhibit amyloidogenesis in the dystrophic neurites of neuritic plaques therefore mitigate this parenchymal amyloid pathology.

As aforementioned, BACE1 plays critical physiological roles in normal neuronal/synaptic structures [60, 61, 63–71, 150–152]. Thus, perhaps the most significant advantage of brain impermeable BACE1 inhibitors over permeable ones is that the former may not elicit much unwanted effect to neurons and synapses in healthy brain regions and structures. Thus, this type of inhibitors might be used chronically as preventive as well as therapeutic regiments. Taken together, brain impermeable BACE1 inhibitors can be expected to have a better target engagement, relative to permeable ones, to act more selectively on the cellular elements exhibiting pathologically enhanced BACE1 activity (i.e., vascular cells and perivascular dystrophic neurites around CAA as well as neuritic plaques). Re-evaluation of some brain impermeable BACE1 inhibitory lead compounds and further development of this class as anti-Aß reagents primarily targeting at CAA but potentially at parenchymal plaques as well, are worth pursuing.

Strategic issues for the development of anti-CAA BACE1 inhibitors also include a more thorough understanding of the biological role of the enzyme in vascular endothelia. So far, no data have shown significant malformation or dysfunction of the cardiovascular system in BACE1 knockout mice (to the best of our knowledge). However, comprehensive in vitro and in vivo investigations would still be needed to firmly confirm that pharmacological inhibition of endothelial BACE1 does not impair vascular health in the central and peripheral systems. Since clinical trials on existing brain permeable BACE1 inhibitors are ongoing and the outcomes could be available in the near future, brain imaging and pathological information about CAA is worth collecting from the subjects participated in the trials. These inhibitors might also have some effect on the vascular cellular components, thus, in addition to the measurements of parenchymal amyloidosis and cognitive performance, clues of improvement on CAA pathology are worth searching from available postmortem brains of the enrolled subjects, even in the case that the cognitive outcomes obtained during the period of clinical trial are not excellent.

## Conclusions

Cerebral vascular health is crucial for brain health in humans. As one of most common brain angiopathic lesions, CAA is associated with vascular deficits but may cause direct and indirect neuronal injury leading to devastating neurological outcomes, therefore playing an unneglectable role for cognitive decline during “normal” brain aging and in AD. CAA has been long regarded as to occur following some primary vascular deficit causing a passive retention of Aß at the brain-blood interface. The findings assembled in this review from original studies point to an active role of increased Aß production by local cellular elements, primarily the endothelial cells and perivascular neuronal terminals, in the pathogenesis of CAA. Such findings encourage the development of active pharmacological approaches to confront the development of CAA. BACE1 is the key enzyme for Aß production. Brain penetrant BACE1 inhibitors are being tested in clinical trials to mitigate plaque development for AD therapy. We propose here brain impermeable BACE1 inhibitors as putative therapeutics to mitigate CAA, which may offer desirable efficacy to prevent vascular amyloidosis yet avoid unwanted side effects duo to inhibition of neuronal and synaptic BACE1 activity in normal brain regions and structures. Attenuating CAA pathogenesis could stabilize vascular integrity and functionality, and improve overall brain health among the elderly at risk of suffering from dementia of the vascular as well as AD types.

## Abbreviations

AD: Alzheimer’s disease
APP: ß-Amyloid precursor protein
Aß: ß-amyloid peptides
BACE1: ß-secretase-1
BBB: Blood brain barrier
CAA: Cerebral amyloid angiopathy
eNOS: Endothelial nitric oxide synthase
PS: Presenilin
β-CTFs: ß-C-terminal fragments
